# Admission Criteria to Paediatric Intensive Care for Oncology Haematology Patients: Updates and Evidence-Based Clinical Recommendations

**DOI:** 10.3390/pediatric18020058

**Published:** 2026-04-14

**Authors:** Ivonne Portaccio, Enzo Picconi, Tony Christian Morena, Giorgio Conti, Marco Piastra

**Affiliations:** 1 Pediatric Intensive Care Unit and Trauma Center, Fondazione Policlinico Universitario “A. Gemelli” IRCCS, Largo Agostino Gemelli 8, 00168 Rome, Italy; 2 Institute of Anesthesia and Intensive Care, Università Cattolica del Sacro Cuore, 00168 Rome, Italy

**Keywords:** paediatric intensive care, paediatric oncology, Phoenix Sepsis Score, non-invasive ventilation, ECMO, CRRT, biomarkers

## Abstract

**Background:** The landscape of paediatric oncology has undergone a remarkable transformation over recent decades. Advances in both oncological and supportive therapies have dramatically improved survival in children with haematological malignancies and solid tumours, with current survival rates exceeding 80% for many childhood cancers. However, this therapeutic success has brought with it an unexpected consequence: the intensification of treatment protocols has led to a parallel increase in life-threatening complications requiring intensive care support. Current evidence indicates that up to 40% of paediatric oncology patients will require admission to a Paediatric Intensive Care Unit (PICU) at some point during their disease trajectory. **Objectives:** This comprehensive review synthesises current evidence to provide an updated framework for PICU admission decision-making in oncology haematology patients. We have integrated the most recently published international guidelines, including the groundbreaking Phoenix 2024 sepsis criteria and the updated PALICC-2 2023 recommendations for paediatric acute respiratory distress syndrome. Beyond establishing admission criteria, we critically analyse the efficacy of advanced support strategies and examine emerging therapeutic approaches in this uniquely vulnerable population. **Methods:** Our methodology encompassed a systematic review of the literature published between 2011 and 2024, complemented by a detailed analysis of current international guidelines and expert consensus statements. We included randomised controlled trials, observational studies, meta-analyses, and consensus conference proceedings specifically addressing the intensive care management of paediatric patients with oncological or haematological conditions. **Main Results:** Several key findings emerge from our analysis. The Phoenix 2024 criteria represent a fundamental reconceptualisation of paediatric sepsis diagnosis, validated through an unprecedented dataset encompassing more than 3 million paediatric encounters. In the realm of respiratory support, early implementation of non-invasive ventilation (NIV) or continuous positive airway pressure (CPAP) has demonstrated remarkable efficacy, reducing the need for invasive mechanical ventilation by 45% (RR 0.45, 95% CI 0.26–0.78) when applied to appropriately selected patients. Extracorporeal membrane oxygenation (ECMO), whilst increasingly utilised, shows survival to decannulation ranging from 52% to 64%, though survival to hospital discharge remains less encouraging at 36–42%. Continuous renal replacement therapy (CRRT) has proven highly effective for tumour lysis syndrome, achieving metabolic correction in 90% of severe cases. Perhaps most promisingly, emerging biomarkers—particularly interleukin-6, interleukin-10, and procalcitonin—have substantially enhanced our ability to stratify infection risk, demonstrating sensitivity exceeding 85% for bacteraemia detection. **Conclusions:** The evidence unequivocally supports several core principles for optimising outcomes in this population. Early identification of deterioration through validated scoring systems enables timely intervention before irreversible organ failure develops. Prompt implementation of non-invasive respiratory support, when appropriately applied, can obviate the need for mechanical ventilation with its attendant complications. Perhaps most critically, centralisation of care in centres with dedicated expertise and comprehensive support capabilities fundamentally improves survival. These findings argue compellingly for the establishment of a formal national network of reference centres, implementing standardised protocols and structured care pathways specifically designed for critically ill paediatric oncology haematology patients.

## 1. Introduction

### 1.1. Epidemiology and Disease Burden

Few achievements in modern medicine rival the dramatic improvement in paediatric cancer survival witnessed over the past five decades. This transformation stands as one of the defining successes of contemporary paediatric medicine. Consider the trajectory: in the 1970s, a diagnosis of childhood cancer carried an approximately 80% probability of death within five years. Today, that equation has fundamentally reversed, with five-year survival rates now reaching 83–90% for many paediatric malignancies [1]. The magnitude of this achievement becomes even more striking when examining specific disease entities. Acute lymphoblastic leukaemia (ALL), the most common childhood cancer accounting for approximately one-quarter of all paediatric malignancies, exemplifies this progress. In high-income countries, cure rates for ALL now approach 90%—a figure that would have seemed impossibly optimistic just a generation ago [2].

This extraordinary success story reflects the convergence of multiple therapeutic advances: intensification of risk-adapted chemotherapy protocols, fundamental improvements in supportive care, steady expansion of indications for haematopoietic stem cell transplantation (HSCT), and, most recently, the introduction of molecularly targeted therapies and revolutionary immunotherapeutic approaches, including chimeric antigen receptor T-cells (CAR-T).

### 1.2. The Paradox of Success: Increasing Need for Intensive Care

Paradoxically, the very therapeutic intensification that has driven improved survival has simultaneously increased the incidence of life-threatening complications. A comprehensive epidemiological analysis conducted across 21 United States jurisdictions, examining trends from 2001 through 2019, has provided crucial insights into the magnitude of this challenge [3]. Between 38% and 40% of paediatric oncology patients now require at least one PICU admission during their treatment course. Whilst oncology haematology patients represent only approximately 3% of total PICU admissions, this proportion has been increasing steadily. Perhaps most sobering, despite substantial therapeutic advances, PICU mortality in this population remains three to four times higher than that observed in the general paediatric intensive care population. Although mortality has declined dramatically—from approximately 35% during the 2000–2010 decade to 11–18% in recent years (2021–2024)—these figures underscore the ongoing vulnerability of critically ill oncology patients.

### 1.3. Identifying Patients at Highest Risk

A landmark multicentre analysis encompassing 10,365 PICU admissions has provided a comprehensive assessment of factors predicting intensive care needs [4]. Disease-related factors exert a profound influence: haematological malignancies confer substantially elevated risk compared to solid tumours (OR 2.4, 95% CI 1.8–3.2, *p* < 0.001), with acute myeloid leukaemia emerging as particularly high-risk. Relapsed or refractory disease more than triples PICU admission risk (OR 3.1, 95% CI 2.3–4.1).

Treatment-related factors constitute another major risk category. Post-HSCT patients, particularly after allogeneic transplantation, face markedly elevated risk (OR 3.5, 95% CI 2.6–4.7). The advent of CAR-T cell therapy has introduced an entirely new category of toxicity, with 40–70% of recipients developing cytokine release syndrome requiring intensive monitoring.

Clinical parameters at assessment provide additional prognostic information: severe neutropaenia below 500 cells per microlitre substantially increases risk (OR 2.8, 95% CI 2.1–3.7), whilst multi-organ dysfunction involving two or more systems dramatically escalates risk (OR 4.5, 95% CI 3.4–5.9). The need for mechanical ventilation carries an odds ratio of 5.2 (95% CI 3.9–6.8), highlighting respiratory failure as both a marker and a driver of critical illness severity.

## 2. PICU Admission Criteria: A Systematic Approach

### 2.1. Foundational Principles

The decision to admit a paediatric oncology haematology patient to intensive care represents one of the most consequential determinations in paediatric medicine. Optimal decision-making requires a nuanced, multidimensional assessment extending well beyond vital signs and laboratory values [5]. Five key domains must be systematically evaluated: objective clinical severity quantified using validated scoring systems, reversibility of the acute condition, overall oncological prognosis and treatment responsiveness, phase within the therapeutic pathway, and crucially, the expressed wishes of the patient and family, including any advance directives.

### 2.2. Validated Assessment Tools

#### 2.2.1. Phoenix Sepsis Score 2024: A New Paradigm

The publication of the Phoenix Sepsis Score in January 2024 represents a fundamental reconceptualisation of paediatric sepsis diagnosis [6,7]. Developed through rigorous modified Delphi methodology and validated on more than 3 million paediatric encounters, the score evaluates dysfunction across four organ systems. The respiratory system contributes 0–3 points (1 point for PaO_2_/FiO_2_ 200–400, 2 points for <200, and 3 points for mechanical ventilation). The cardiovascular system, carrying the greatest prognostic weight, contributes up to 6 points: 2 points each for age-specific severe hypotension, lactate > 5 mmol/L, and any vasopressor requirement. The coagulation system adds 1 point for platelets < 100,000/μL and 1 point for INR > 1.3 or D-dimer > 2 mg/L FEU. The neurological system assigns 2 points for GCS ≤ 10.

Age-specific hypotension thresholds are: systolic blood pressure < 60 mmHg (neonates), <70 mmHg (1 month–1 year), <70 + (age × 2) mmHg (1–10 years), and <90 mmHg (>10 years). Paediatric sepsis is defined as suspected/confirmed infection plus Phoenix Score ≥ 2; septic shock requires sepsis plus ≥ 1 cardiovascular point.

The advantages prove substantial and have been extensively discussed in the recent literature [8,9]: approximately 40% reduction in false positive diagnoses compared to SIRS criteria, improved correlation with mortality (AUC 0.81 versus 0.71), robust validation across diverse international populations, and crucially, applicability even in resource-limited settings. This represents a genuine paradigm shift rather than incremental refinement.

#### 2.2.2. O-PRISM Score: Oncology-Specific Risk Assessment

The Oncological Paediatric Risk of Mortality score (O-PRISM) adapts PRISM III specifically for oncology patients [10]. Beyond standard PRISM parameters, it incorporates three oncology-specific elements: absolute neutropaenia < 500/μL (2 points), post-HSCT status (3 points), and severe mucositis grade ≥ 3 (2 points). The score demonstrates excellent discriminative ability with an AUC-ROC of 0.89 (95% CI 0.85–0.93), substantially superior to non-oncology-specific scores in this population.

### 2.3. Organ-Specific Admission Criteria

Contemporary evidence supports systematic, organ-based admission criteria, as detailed in Table 1, whilst acknowledging that clinical judgement remains essential when multiple systems show borderline dysfunction or trajectory suggests rapid deterioration. Criteria were derived from the Phoenix 2024 paediatric sepsis consensus [7,8,9], the PALICC-2 2023 guidelines for paediatric ARDS [11], the ACCCM 2017 haemodynamic support parameters [12], and standard expert consensus thresholds for renal, neurological, and haematological dysfunction as referenced throughout the text.

## 3. Management of Respiratory Failure

### 3.1. Understanding the Aetiology

Acute respiratory failure stands as the single most common reason for PICU admission among paediatric oncology haematology patients, accounting for approximately 45–50% of admissions [13]. The aetiological landscape proves remarkably complex, frequently involving multiple concurrent processes. Infectious causes predominate (60–70%): bacterial pneumonias account for 30% (*Pseudomonas aeruginosa*, *Staphylococcus aureus*, and Enterobacteriaceae), viral pneumonias 25% (RSV, influenza, parainfluenza, and SARS-CoV-2), and fungal infections 15% (*Aspergillus*, *Pneumocystis*, and *Candida*). Non-infectious causes (30–40%) encompass cardiogenic and non-cardiogenic pulmonary oedema, diffuse alveolar haemorrhage, leukaemic/lymphomatous infiltration, drug toxicity (bleomycin, methotrexate, and busulfan), idiopathic interstitial pneumonia post-HSCT, and engraftment syndrome.

### 3.2. Non-Invasive Ventilation: The Evidence Base

The seminal work by Squadrone and colleagues in immunocompromised haematological patients demonstrated that early CPAP application within 8 h of acute respiratory failure onset yields multiple significant benefits [14]: prevention of progression to ARDS (12% versus 33%, *p* = 0.002), dramatic reduction in intubation need (RR 0.45, 95% CI 0.26–0.78), and improved ICU survival (77% versus 61%, *p* = 0.04). These findings strongly support aggressive early application of non-invasive respiratory support. Children with haematology/oncology diagnoses or immunosuppression complicated by ARDS can undergo non-invasive ventilation through several interfaces [15], demonstrating a clinically significant outcome difference: the high possibility of NIV failure compared to previously healthy patients does not preclude its use; on the contrary, an early NIV trial is indicated in all patients with malignancy and respiratory failure [16].

The updated PALICC-2 guidelines (2023) provide comprehensive recommendations for NIV/CPAP implementation [11]. Appropriate candidates include patients with PaO_2_/FiO_2_ ratios of 200–300 who maintain haemodynamic stability, remain sufficiently alert (GCS > 10), demonstrate effective cough with manageable secretions, and have no contraindications. The implementation protocol emphasises oro-nasal masks or helmet interfaces for better tolerance, conservative initial settings (CPAP 5–8 cmH_2_O incrementing by 2 cmH_2_O to maximum 12; BiPAP with IPAP 10–12 cmH_2_O, EPAP 5–6 cmH_2_O), physiological targets (SpO_2_ 92–97%, respiratory rate < 30% above baseline), and close monitoring with clinical reassessment every 30 min for the first 2 h.

Clinicians must maintain a low threshold for recognising NIV failure: lack of PaO_2_/FiO_2_ improvement within 1–2 h, progressive neurological deterioration (GCS < 10), haemodynamic instability, or paradoxically increased work of breathing all mandate transition to invasive ventilation. Both increased physiological scores (such as PRISM-III or PELOD) and failing organs have been correlated to NIV failure [17].

In summary, in haematological children, NIV is best employed early, in carefully selected patients, and under strict monitoring. A high failure rate ensues if applied too late, with the attendant risk of delaying necessary intubation in severe ARDS. Close monitoring and an experienced team are therefore essential prerequisites (Table 2).

### 3.3. Invasive Mechanical Ventilation: When Prevention Fails

When non-invasive ventilation fails or proves contraindicated, lung-protective ventilation strategies represent a mandatory standard of care [20]. Contemporary PALICC-2 recommendations specify: tidal volumes 5–8 mL/kg ideal body weight (3–6 mL/kg if severe ARDS), plateau pressure ≤ 28 cmH_2_O (≤32 if reduced chest compliance), driving pressure ≤ 15 cmH_2_O, individualised PEEP titration, inspired oxygen minimised for SpO_2_ 88–92% in ARDS (92–97% if not ARDS), and permissive hypercapnia provided pH > 7.15–7.20.

For severe ARDS refractory to optimal conventional ventilation, rescue interventions include: prone positioning ≥12 h daily if PaO_2_/FiO_2_ < 150, recruitment manoeuvres (controversial, consider if atelectasis), inhaled nitric oxide (may improve oxygenation but not mortality), high-frequency oscillatory ventilation [21,22], and ECMO if PaO_2_/FiO_2_ < 60–80 despite optimisation [23].

## 4. Septic Shock Management According to Phoenix 2024 Criteria

### 4.1. Implementation of the Golden Hour Bundle

The Surviving Sepsis Campaign paediatric guidelines emphasise the critical first hour following sepsis recognition through six essential interventions with specific time targets [24]. These reflect the urgency that sepsis demands, with evidence demonstrating that delays in key interventions lead inexorably to worse outcomes.

Whilst 60 min represents the outer time limit for antibiotic administration, clinicians should strive for even more rapid delivery when feasible, as each hour of delay increases mortality. First fluid bolus ideally should be infused over 5–10 min. Lactate trajectory over subsequent hours—particularly failure to decline—predicts poor outcomes more powerfully than a single elevated value (Table 3).

### 4.2. Antimicrobial Selection in the Neutropaenic Patient

Antibiotic selection requires careful consideration of local resistance patterns, individual risk factors, clinical severity, and the presence of indwelling devices. Table 4 provides a framework stratified by clinical severity.

### 4.3. Advanced Haemodynamic Management

In shock refractory to initial fluid resuscitation, invasive haemodynamic monitoring becomes essential [12]. Central venous oxygen saturation (ScvO_2_) targeting >70% predicts outcomes with reasonable accuracy (sensitivity 77% and specificity 85%). Cardiac index should be maintained >3.3 L/min/m^2^ via echocardiography or thermodilution. Respiratory variation indices (PPV/SVV) predict fluid responsiveness when >13%, superior to static measures for determining whether additional fluid will augment cardiac output versus causing harmful overload. Lactate clearance > 10% every 2 h has emerged as perhaps the single most powerful predictor of survival.

Vasopressor selection should follow systematic escalation guided by haemodynamic state. First-line therapy consists of adrenaline (0.05–0.3 μg/kg/min) or noradrenaline (0.05–0.5 μg/kg/min). In cold shock (↑SVR and ↓CO), add milrinone (0.25–0.75 μg/kg/min) for inotropic support and afterload reduction. In warm shock (↓SVR and ↑CO), escalate noradrenaline up to 2 μg/kg/min or add vasopressin (0.0003–0.002 U/kg/min). For refractory shock, consider hydrocortisone (1–2 mg/kg every 6 h, maximum 50 mg), though this remains controversial.

### 4.4. Biomarkers: Enhancing Diagnostic Precision

Recent meta-analyses have established procalcitonin as superior to C-reactive protein for both diagnostic and prognostic purposes [25,26]. Specific cut-offs provide clinically actionable information: PCT < 0.5 ng/mL makes bacterial infection unlikely (NPV 93%), 0.5–2 ng/mL suggests possible localised infection, >2 ng/mL indicates probable systemic infection (PPV 85%), and >10 ng/mL strongly suggests septic shock (specificity 95%). Procalcitonin kinetics enhance interpretation: reduction > 50% within 48 h indicates good therapeutic response, whilst failure to decline suggests inadequate antibiotic selection or uncontrolled focus.

Cytokine measurements show considerable promise for risk stratification [27,28,29]. Individual cytokines demonstrate good performance: IL-6 > 60 pg/mL predicts bacteraemia with 82% sensitivity and 78% specificity (AUC 0.85), whilst IL-10 > 20 pg/mL predicts septic shock with 77% sensitivity and 79% specificity (AUC 0.87). However, combining biomarkers yields superior results: IL-6 plus IL-10 achieves 91% sensitivity and 85% specificity for bacteraemia (AUC 0.92), whilst PCT plus IL-10 (thresholds 0.425 ng/mL and 4.37 pg/mL) attains 100% sensitivity with 89% specificity (AUC 0.95). In CAR-T therapy, IL-2 > 100 U/mL predicts severe CRS with 85% sensitivity and 82% specificity (AUC 0.88).

Presepsin (soluble CD14-ST) represents an emerging biomarker particularly suited to neutropaenic patients [30]. Unlike many markers, presepsin levels remain uninfluenced by leucocyte count, avoiding a major confounding factor in the neutropaenic host. Presepsin peaks earlier than procalcitonin (2–3 h versus 12–24 h), potentially allowing faster clinical decision-making. At a cut-off of 600 pg/mL, presepsin demonstrates 87% sensitivity and 81% specificity for sepsis in neutropaenic patients. Two independent paediatric studies published in 2023, by Cerasi et al. [30], have both documented significantly elevated presepsin levels in children with oncological and haematological diseases experiencing febrile neutropaenia compared with healthy controls, supporting its potential role in early infection stratification in this high-risk population.

Finally, pancreatic stone protein (PSP) represents a further emerging candidate with potential advantages in the oncology haematology setting. PSP rises earlier than conventional acute-phase reactants and demonstrates higher specificity for bacterial infection, though paediatric data in immunocompromised patients remain limited and prospective validation in this population is needed.

## 5. Extracorporeal Support Therapies

### 5.1. ECMO: Expanding Indications and Evolving Outcomes

ECMO utilisation in paediatric oncology haematology patients has increased approximately threefold over the past decade [23]. A recent multicentre study of 149 patients (2009–2021) documented that isolated respiratory failure constitutes the most common indication (46%), followed by combined cardio-respiratory failure (28%), isolated cardiac failure (25%), and, rarely, extracorporeal CPR (1%).

Patient selection requires careful consideration of both severity and prognosis. For respiratory indications (VV-ECMO), appropriate candidates demonstrate oxygenation index > 40 for >4 h, PaO_2_/FiO_2_ < 60 on optimal ventilation, or pH < 7.20 from hypercapnia despite maximal ventilation. For cardiac indications (VA-ECMO), criteria include cardiac index < 2.0 L/min/m^2^ despite maximal support, lactate > 4 mmol/L and rising, or ScvO_2_ < 55% despite optimisation.

Understanding outcomes requires attention to multiple time points, as survival attenuates considerably from decannulation to discharge to long-term follow-up, as shown in Table 5.

Multivariate analysis identified independent negative prognostic factors: pre-ECMO CPR (OR 3.0, 95% CI 1.2–7.7), renal dysfunction requiring CRRT (OR 2.8, 95% CI 1.4–5.6), invasive fungal infection (OR 4.2, 95% CI 1.8–9.7), and ECMO duration > 14 days (OR 3.5, 95% CI 1.6–7.8).

Oncology patients experience substantially higher complication rates than general PICU populations [33]: ECMO-related infections (25% versus 12%, *p* = 0.001), major bleeding (42% versus 28%, *p* = 0.01), circuit thrombosis (18% versus 12%, NS), and neurological complications (15% versus 8%, *p* = 0.03).

### 5.2. CRRT: Beyond Simple Renal Replacement

CRRT serves multiple critical functions in oncology haematology patients extending well beyond renal replacement [34,35]. Indications include severe tumour lysis syndrome, acute kidney injury with fluid overload > 10%, methotrexate toxicity (MTX > 10 μmol/L at 42 h), transplant-associated thrombotic microangiopathy, cytokine removal in severe CRS or macrophage activation syndrome, and metabolic support in hyperammonaemia or severe lactic acidosis.

Tumour lysis syndrome, whilst occurring in only approximately 9% of patients with paediatric cancer, represents a true oncological emergency [36]. When CRRT becomes necessary, a specific protocol optimises outcomes: CVVHDF modality (combining convection and diffusion), dialysis dose 50 mL/kg/h (higher than standard 25–35), treatment duration 48–72 h, with metabolic targets of uric acid < 6 mg/dL within 6 h, phosphorus < 4.5 mg/dL within 12 h, and potassium < 5 mEq/L within 2 h.

Outcomes have proven remarkably encouraging: resolution of metabolic abnormalities in essentially 100% of cases within 12 h, renal function recovery in >90% of survivors, need for chronic dialysis < 2%, and TLS-related mortality reduced from approximately 40% in the pre-CRRT era to just 5% currently.

Appropriate CRRT prescription requires attention to multiple technical parameters. Modality selection should favour CVVHDF for hypermetabolic states, whilst CVVH or CVVHD may suffice for standard indications. Dialysis dose should be 25–35 mL/kg/h for standard prescriptions, but increased to 40–60 mL/kg/h in hypercatabolic conditions. Blood flow rates should be 3–5 mL/kg/min for standard prescriptions, but increased to 5–8 mL/kg/min (maximum 10) for metabolic emergencies. Regional citrate anticoagulation is strongly preferred when platelets are <50,000/μL, given the high bleeding risk. Fluid balance targets should be net negative 0.5–1 mL/kg/h once haemodynamic stability is assured.

### 5.3. Therapeutic Apheresis

Several conditions benefit from therapeutic plasma exchange or apheresis [37]. Transplant-associated thrombotic microangiopathy post-HSCT typically requires 5–7 sessions (1.5× plasma volume per session), achieving survival rates of 50–60%. Severe CAR-T CRS may benefit from 1 to 3 sessions, reducing IL-6 levels by >80%. Hyperviscosity syndrome requires plasma exchange targeting viscosity < 4 centipoise. Cryoglobulinaemia requires circuit warming to 37 °C to prevent cryoprecipitation.

## 6. Special Populations and Novel Therapies

### 6.1. The Post-HSCT Patient: Time-Dependent Complications

Post-transplant patients present with highly time-dependent complications varying systematically by time elapsed since transplant [38], as detailed in Table 6. Incidence ranges derived from: Corbacioglu et al. [38] (VOD/SOS), Jodele et al. [37] (TA-TMA), Lee et al. [39] (CRS), and Rowan et al./Tamburro et al. [13,19] (engraftment/early post-HSCT complications).

### 6.2. CAR-T Cell Therapy: Novel Toxicities

CAR-T therapy has revolutionised the treatment of certain relapsed/refractory haematological malignancies but introduces unique toxicities requiring specialised intensive care management [39,40].

#### 6.2.1. Cytokine Release Syndrome

The ASTCT consensus grading provides standardised severity assessment: Grade 1 (fever ≥ 38 °C only), Grade 2 (plus fluid-responsive hypotension or O_2_ < 40%), Grade 3 (plus vasopressors or O_2_ > 40%), Grade 4 (plus multiple vasopressors or mechanical ventilation), and Grade 5 (death).

Management escalates systematically. Grade 1 requires only supportive care with paracetamol and close monitoring. Grade 2 mandates tocilizumab (8 mg/kg, maximum 800 mg) combined with aggressive fluids. Grades 3–4 require tocilizumab every 8 h for up to three doses, dexamethasone (10 mg/m^2^ every 6 h), full PICU support, including vasopressors and mechanical ventilation, and consideration of second-line agents (siltuximab and anakinra) if refractory.

#### 6.2.2. Neurotoxicity (ICANS)

ICANS grading utilises the ICE score (0–10 points evaluating orientation, naming, commands, and writing): Grade 1 (ICE 7–9), Grade 2 (ICE 3–6), Grade 3 (ICE 0–2), and Grade 4 (plus status epilepticus, cerebral oedema, or coma).

Management differs fundamentally from CRS. Grades 1–2: dexamethasone (10 mg every 6 h) plus prophylactic levetiracetam. Grades 3–4: high-dose methylprednisolone (1 gramme per day × 3), intubation if GCS < 8, ICP monitoring if oedema present, and critically, avoid tocilizumab, which may worsen neurotoxicity.

### 6.3. Emerging Therapeutic Approaches

Several biological agents targeting specific inflammatory pathways are under investigation [41]. Anakinra (anti-IL-1) is being studied at 2–10 mg/kg per day for MAS/HLH. Emapalumab (anti-IFNγ) shows promise at 1 mg/kg in refractory HLH. Ruxolitinib (JAK inhibitor) is being evaluated at 5–10 mg/m^2^ twice daily for CRS/GVHD.

Novel extracorporeal cytokine adsorption devices aim to remove inflammatory mediators [42]. CytoSorb demonstrates >70% IL-6 reduction within 24 h. The oXiris haemofilter combines CRRT with endotoxin adsorption. The Seraph-100 filter aims to remove pathogens directly from blood, though paediatric experience remains limited.

## 7. Organisational Models and Implementation

### 7.1. The Case for Centralisation: Hub and Spoke Model

Accumulating evidence strongly supports centralisation of paediatric oncology intensive care in specialised centres [43]. Effective hub centres require several essential capabilities: PICU with ≥12 dedicated beds, a multidisciplinary team (intensivists, oncologists, and nephrologists) available 24/7, immediate availability of ECMO, CRRT, and therapeutic apheresis, a 24 h laboratory with flow cytometry, a blood bank providing irradiated/leucodepleted products, and protocols specifically designed for immunocompromised patients.

The benefits prove substantial and statistically robust: mortality decreases from 23% to 15% (*p* < 0.001), representing approximately 35% relative mortality reduction; length of stay decreases from 8.2 to 6.4 days (*p* = 0.02); and most compellingly, one-year survival increases from 52% to 68% (*p* < 0.001), demonstrating that centralised care benefits extend beyond initial hospitalisation.

### 7.2. Early Warning Systems and Fast-Track Protocols

Implementation of systematic early warning systems has demonstrated clear benefits [44]. The Paediatric Oncology Warning Score (POWS) incorporates heart rate, respiratory rate, blood pressure, oxygen saturation, mental status, and temperature. When scores reach ≥4, immediate rapid response team activation occurs. The system achieves 89% sensitivity and 76% specificity for predicting 24 h deterioration, with implementation associated with dramatic cardiac arrest reduction from 2.3 to 0.8 per 1000 patient days.

Standardised fast-track protocols for common critical presentations substantially reduce time to key interventions. For febrile neutropaenia (temperature > 38.3 °C or >38 °C × 1 h with ANC < 500), the protocol mandates blood cultures, intravenous antibiotics, and fluid resuscitation within 30 min, achieving 30% mortality reduction. For suspected sepsis (Phoenix ≥ 2 plus suspected infection), complete bundle and PICU activation within 30 min reduces time to antibiotics by 50%. For tumour lysis syndrome (K > 6, *p* > 6.5, uric acid > 8, or creatinine doubling), rasburicase, hyperhydration, and nephrology consultation within 30 min reduce dialysis requirements by 40%. For respiratory distress (RR > 30% baseline plus SpO_2_ < 92%), CPAP/NIV initiation, PICU notification, and chest radiography within 30 min reduce intubations by 35%.

## 8. Outcomes and Prognosis

### 8.1. The Trajectory of Improvement

Analysis of mortality trends reveals substantial continuing improvement, as shown in Table 7. Historical mortality data derived from: Tamburro et al. [13] (1996–2004 period), Wösten-van Asperen et al. [45] (systematic review), Pillon et al. [46] (2010–2019 period), and Soeteman et al. [47] (2021–2024 period).

This progressive improvement reflects cumulative advances: establishment of dedicated PICUs in the 1990s, systematic sepsis protocols and NIV in the 2000s, expanded ECMO/CRRT utilisation with targeted therapies in the 2010s, and most recently, CAR-T therapies with precision medicine approaches [46,47].

### 8.2. Prognostic Factors

Large-scale multivariate analysis of >10,000 patients identified key independent predictors [45]. Factors substantially increasing mortality: mechanical ventilation requirement (OR 4.8, 95% CI 3.6–6.4), multi-organ dysfunction ≥ 3 organs (OR 5.2, 95% CI 3.9–6.9), post-allogeneic HSCT (OR 2.9, 95% CI 2.1–4.0), invasive fungal infection (OR 3.4, 95% CI 2.3–5.0), and delayed PICU admission > 24 h (OR 2.1, 95% CI 1.5–2.9).

Protective factors include: admission at diagnosis pre-chemotherapy (OR 0.4, 95% CI 0.2–0.7), high-volume centre managing >50 cases per year (OR 0.6, 95% CI 0.4–0.8), and complete sepsis bundle application (OR 0.5, 95% CI 0.3–0.7).

### 8.3. Beyond Survival: Long-Term Outcomes

Longitudinal follow-up at two years post-PICU discharge provides a crucial perspective [45,48]. Encouragingly, 72% of survivors return to school/age-appropriate activities, and 65% achieve PedsQL scores > 70. However, 35% require ongoing rehabilitation, 22% demonstrate neurocognitive deficits, and post-traumatic stress disorder affects 18% of survivors and 32% of families, emphasising the importance of comprehensive follow-up extending beyond hospital discharge.

## 9. Clinical Practice Recommendations

### 9.1. Grade A Recommendations (Strong Evidence from RCTs/Meta-Analyses)

1. Use Phoenix 2024 criteria for sepsis diagnosis (Level 1a) [6,7]
2. Administer antibiotics within 60 min in suspected sepsis (Level 1a) [24]
3. Apply protective ventilation with tidal volumes 5–8 mL/kg (Level 1a) [20]
4. Initiate early CRRT if fluid overload > 10% (Level 1b) [34]
5. Use tocilizumab for CAR-T CRS grade ≥ 2 (Level 1b) [39]

### 9.2. Grade B Recommendations (Moderate Evidence from Observational Studies)

1. Trial NIV/CPAP before intubation if PaO_2_/FiO_2_ 200–300 (Level 2a) [11,14]
2. Implement invasive haemodynamic monitoring in refractory shock (Level 2b) [12]
3. Consider ECMO in selected patients with good oncological prognosis (Level 2b) [23,31]
4. Utilise PCT and cytokines to guide antibiotic therapy (Level 2a) [25,26,27,28]
5. Centralise care in PICUs with oncological expertise (Level 2b) [43]

### 9.3. Grade C Recommendations (Expert Consensus)

1. Reserve corticosteroids for catecholamine-refractory shock (Level 3) [24]
2. Initiate early defibrotide in suspected VOD/SOS (Level 3) [38]
3. Target glycaemia 140–180 mg/dL in critically ill patients (Level 3) [24]
4. Encourage early mobilisation when haemodynamically stable (Level 4)
5. Provide structured psychological support to patients and families (Level 4)

## 10. Conclusions and Future Perspectives

### 10.1. Key Messages

This comprehensive evidence synthesis yields five key messages. First, Phoenix 2024 criteria represent a genuine paradigm shift, offering substantially improved specificity and prognostic correlation whilst maintaining practical applicability. Second, early non-invasive ventilation can prevent up to 45% of intubations when appropriately applied. Third, extracorporeal support therapies have evolved into established modalities with well-defined indications, achieving ECMO discharge survival of 36–42% and CRRT resolution of tumour lysis syndrome in >90% of cases. Fourth, multiparametric biomarkers substantially improve risk stratification. Fifth, centralisation in high-volume centres reduces mortality by 30–40%.

### 10.2. Future Directions

Priority research areas include: validation of predictive scores for specific subpopulations (CAR-T and HSCT), randomised trials on optimal timing for ECMO/CRRT, biomarkers distinguishing infection from inflammatory syndromes, and neuroprotection strategies in CAR-T neurotoxicity. Technological innovations promise transformation: machine learning algorithms for deterioration prediction, miniaturised ECMO for patients < 10 kg, continuous cytokine biosensors, and expanded telemedicine infrastructure. Organisational development should focus on: creating formal national networks of reference centres, implementing national registries capturing all oncology PICU admissions, developing specific training pathways, and establishing evidence-based national guidelines.

### 10.3. Call to Action

We issue an urgent call for: (1) establishment of a National Paediatric Oncology Intensive Care Network with formal designation of reference centres, clear referral pathways, telemedicine consultation, education programmes, and research infrastructure; (2) standardisation of management protocols through consensus development, wide dissemination, and monitoring of adherence; (3) universal implementation of early warning systems with professional society endorsement and mandatory adoption; (4) continuous professional development through structured educational programmes and competency verification; and (5) active promotion of multicentre research through dedicated funding, research consortia, streamlined regulatory processes, and data sharing agreements.

Only through this comprehensive, systematic approach will we continue improving outcomes for these vulnerable patients, ensuring appropriate resource utilisation, compassionate communication, and comprehensive psychosocial support throughout their care pathway.

## Figures and Tables

**Table 1:** Updated PICU Admission Criteria for Organ Dysfunction.

System	Admission Criteria	Intervention Timing	Monitoring
**Respiratory**	RR > 30% from baseline SpO_2_ < 92% in room air PaO_2_/FiO_2_ < 300 FiO_2_ need > 0.4 Moderate-severe respiratory distress	NIV/CPAP within 2 h if PaO_2_/FiO_2_ 200–300	Blood gases every 1–2 h Chest X-ray every 12 h Lung POCUS
**Cardiovascular**	Hypotension (Phoenix criteria) Lactate > 5 mmol/L ScvO_2_ < 65% Oliguria < 0.5 mL/kg/h × 2 h Vasopressor requirement	Fluids 20 mL/kg in 20 min Vasopressors if non-responder Age-appropriate MAP target	Invasive BP monitoring Continuous ScvO_2_ Echocardiography every 24 h
**Neurological**	GCS < 12 or decrease ≥ 3 points Status epilepticus Signs of Increased ICP Suspected PRES/ICANS	Neuroprotection IV antiepileptics Consider intubation if GCS < 8	Continuous EEG Pupillometry Urgent CT/MRI
**Renal**	Fluid overload > 10% K^+^ > 6.5 mEq/L Metabolic acidosis pH < 7.2 Anuria > 6 h Creatinine × 3 baseline	Diuretic trial CRRT if FO > 15% or severe electrolyte imbalance	Hourly fluid balance Electrolytes every 4–6 h Renal POCUS
**Haematological**	Febrile neutropaenia + instability Clinical DIC Severe TLS Major haemorrhage Hb < 7 g/dL + instability	Antibiotics within 60 min Transfusion support Rasburicase if TLS	CBC every 6 h Coagulation every 8 h Uric acid, P, K every 4 h if TLS
**Metabolic**	pH < 7.20 (non-respiratory) Lactate > 10 mmol/L Symptomatic hypoglycaemia Hyperammonaemia > 200 μg/dL	Correct cause CRRT if indicated Glycaemic target 140–180 mg/dL	ABG every 2–4 h Glucose every 1–2 h Ammonia every 6 h

PRES: posterior reversible encephalopathy syndrome; ICANS: immune effector cell-associated neurotoxicity syndrome; TLS: tumour lysis syndrome; FO: fluid overload.

**Table 2:** Predictors of NIV Success/Failure in Oncology Haematology Patients.

Factor	NIV Success	NIV Failure	*p*-Value	Ref.
Initial PaO_2_/FiO_2_	245 ± 45	142 ± 38	<0.001	[18]
SOFA score	4.2 ± 1.8	8.6 ± 2.4	<0.001	[18]
Neutrophils/μL	>500	<100	0.002	[16]
Infiltrate type	Interstitial	Diffuse alveolar	0.01	[16]
Aetiology	Pulmonary oedema	ARDS/pneumonia	0.03	[19]
Lactate (mmol/L)	<2	>4	<0.001	[19]

**Table 3:** First Hour Bundle in Paediatric Sepsis.

Intervention	Timing	Details	Evidence
1. Vascular access	<5 min	2 peripheral IV or IO if difficulty	Strong
2. Microbiological samples	<10 min	Blood cultures × 2, urine culture, other sites	Strong
3. Antibiotics	<60 min	Empirical broad-spectrum	Strong
4. Fluid resuscitation	<20 min	Crystalloids 20 mL/kg, repeatable × 3	Strong
5. Lactate measurement	<30 min	Target < 2 mmol/L within 6 h	Moderate
6. Vasopressors	<60 min	If shock persists after 40–60 mL/kg fluids	Strong

**Table 4:** Empirical Antibiotic Therapy in Febrile Neutropaenia.

Clinical Scenario	First Line	Alternatives	Notes
**Stable febrile neutropaenia**	Piperacillin-tazobactam 100 mg/kg every 6 h or Cefepime 50 mg/kg every 8 h	Meropenem 20 mg/kg every 8 h	Monotherapy sufficient
**Sepsis without shock**	Piperacillin-tazobactam + Amikacin 15 mg/kg per day	Meropenem + Vancomycin	Consider vancomycin if CVC
**Septic shock**	Meropenem 40 mg/kg every 8 h + Vancomycin 15 mg/kg every 6 h + Amikacin 20 mg/kg per day	Consider: Caspofungin, Voriconazole, Colistin (if MDR)	Combination therapy mandatory
**Suspected fungal**	Add: Caspofungin 70 mg/m^2^ Day 1, then 50 mg/m^2^ per day	Liposomal AmB 3–5 mg/kg per day	If Aspergillus: voriconazole

**Table 5:** ECMO Outcomes in Paediatric Oncology Haematology Patients.

Population	N	Survival to Decannulation	Survival to Discharge	1-Year Survival	Ref.
Oncology (all)	118	52% (62/118)	36% (43/118)	28%	[23]
Post-HSCT	31	64% (20/31)	42% (13/31)	32%	[23]
Leukaemia/lymphoma	67	48%	31%	22%	[31]
Solid tumours	51	59%	45%	38%	[31]
CAR-T CRS	12	75%	58%	50%	[32]

**Table 6:** Post-HSCT Complications and PICU Management.

Period	Complication	Incidence	Specific Management
**Pre-engraftment** (D0–30)	Engraftment syndrome	15–20%	Corticosteroids, respiratory support
	VOD/SOS	10–15%	Defibrotide 25 mg/kg per day, strict fluid balance
	Severe mucositis	40–60%	Parenteral nutrition, opioid analgesia
	CRS	20–30%	Tocilizumab, corticosteroids
**Early post-engraftment** (D30–100)	Acute GVHD	30–50%	Methylprednisolone 2 mg/kg, tacrolimus
	CMV/EBV reactivation	20–40%	Ganciclovir, rituximab if PTLD
	TA-TMA	10–30%	Eculizumab, plasma exchange, CRRT
**Late** (>D100)	Chronic GVHD	30–70%	Multimodal immunosuppression
	Interstitial pneumonia	5–15%	High-dose corticosteroids, etanercept
	BOOP/COP	2–5%	Corticosteroids, macrolides

VOD: veno-occlusive disease; SOS: sinusoidal obstruction syndrome; GVHD: graft-versus-host disease; TA-TMA: transplant-associated thrombotic microangiopathy; BOOP: bronchiolitis obliterans organising pneumonia.

**Table 7:** Evolution of PICU Mortality in Oncology Haematology Patients.

Period	PICU Mortality	Hospital Mortality	1-Year Survival	Key Drivers
1990–2000	40–50%	55–65%	25–35%	Dedicated PICUs
2001–2010	27–35%	35–45%	35–45%	Sepsis protocols, NIV
2011–2020	18–23%	25–30%	45–55%	ECMO, CRRT, targeted therapy
2021–2024	11–18%	18–25%	55–65%	CAR-T, precision medicine

## Data Availability

No new data were created or analysed in this study.
